# Supplementary material for: “Wanting” versus “needing” related value: An fMRI meta‐analysis
Source: Brain Behav. 2022 Aug 24;12(9):e32713. doi: 10.1002/brb3.2713 (PMC9480935; doi:10.1002/brb3.2713)
Supplement: Supplementary file 1 — Supplementary material [file BRB3-12-e32713-s001.docx]

**PRISMA for Wanting_ST_**

Studies included in quantitative synthesis (meta-analysis)
(n = 22)

Studies included in qualitative synthesis
(n = 22)

Full-text articles excluded, with reasons
(n = 7)

Full-text articles assessed for eligibility
(n = 29)

Records excluded
(n = 134)

Records screened
(n = 163)

Records after duplicates removed
(n = 163)

Additional records identified through other sources
(n = 4)

## Identification

## Eligibility

## Included

## Screening

Records identified through database searching
(n = 159)

**PRISMA Needing_ST_**

Studies included in quantitative synthesis (meta-analysis)
(n = 16)

Studies included in qualitative synthesis
(n = 16)

Full-text articles excluded, with reasons
(n = 25)

Full-text articles assessed for eligibility
(n = 41)

Records excluded
(n = 388)

Records screened
(n = 429)

Records after duplicates removed
(n = 429)

Additional records identified through other sources
(n = 53)

## Identification

## Eligibility

## Included

## Screening

Records identified through database searching
(n = 376)

**Leave one experiment out (LOEO) analysis**

**WANTING_ST_**

Threshold

-- p value = 1

-- intensity = -1.633123935319537e+16

-- cluster size = 5

Number of clusters found: 10

----------------------

Cluster 1

Number of voxels: 354

Peak MNI coordinate: 8 -26 -22

Peak MNI coordinate region: // Right Brainstem // Midbrain // undefined // undefined // undefined // undefined

Peak intensity: 1

# voxels structure

354 --TOTAL # VOXELS--

354 Midbrain

199 Left Brainstem

155 Right Brainstem

12 Gray Matter

6 Substania Nigra

6 Red Nucleus

----------------------

Cluster 2

Number of voxels: 527

Peak MNI coordinate: 18 4 -12

Peak MNI coordinate region: // Right Cerebrum // Sub-lobar // Lentiform Nucleus // Gray Matter // Putamen // undefined

Peak intensity: 1

# voxels structure

527 --TOTAL # VOXELS--

527 Right Cerebrum

523 Sub-lobar

443 Gray Matter

244 Lentiform Nucleus

210 Putamen

204 Caudate_R (aal)

171 Caudate

131 Putamen_R (aal)

126 Caudate Head

81 White Matter

77 Extra-Nuclear

77 Pallidum_R (aal)

45 Caudate Body

31 Lateral Globus Pallidus

3 Medial Globus Pallidus

3 Frontal Lobe

3 Subcallosal Gyrus

3 Lateral Ventricle

3 Cerebro-Spinal Fluid

1 Limbic Lobe

1 Anterior Cingulate

----------------------

Cluster 3

Number of voxels: 480

Peak MNI coordinate: -4 10 -12

Peak MNI coordinate region: // Left Cerebrum // Frontal Lobe // Subcallosal Gyrus // Gray Matter // brodmann area 25 // Caudate_L (aal)

Peak intensity: 1

# voxels structure

480 --TOTAL # VOXELS--

480 Left Cerebrum

455 Sub-lobar

336 Gray Matter

195 Lentiform Nucleus

184 Putamen_L (aal)

180 Caudate_L (aal)

177 Putamen

144 White Matter

125 Extra-Nuclear

110 Caudate

84 Caudate Head

45 Pallidum_L (aal)

26 Caudate Body

16 Lateral Globus Pallidus

14 Frontal Lobe

14 Subcallosal Gyrus

11 Limbic Lobe

10 Anterior Cingulate

3 brodmann area 25

2 brodmann area 13

1 Medial Globus Pallidus

----------------------

Cluster 4

Number of voxels: 173

Peak MNI coordinate: 32 22 -6

Peak MNI coordinate region: // Right Cerebrum // Frontal Lobe // Inferior Frontal Gyrus // White Matter // undefined // undefined

Peak intensity: 1

# voxels structure

173 --TOTAL # VOXELS--

173 Right Cerebrum

112 Insula_R (aal)

105 White Matter

105 Sub-lobar

68 Frontal Lobe

60 Inferior Frontal Gyrus

60 Gray Matter

59 Insula

40 Extra-Nuclear

24 brodmann area 13

19 brodmann area 47

11 Sub-Gyral

8 brodmann area 45

5 Putamen_R (aal)

3 Claustrum

1 Frontal_Inf_Tri_R (aal)

----------------------

Cluster 5

Number of voxels: 174

Peak MNI coordinate: -32 16 -2

Peak MNI coordinate region: // Left Cerebrum // Sub-lobar // Insula // Gray Matter // brodmann area 47 // Insula_L (aal)

Peak intensity: 1

# voxels structure

174 --TOTAL # VOXELS--

174 Left Cerebrum

169 Sub-lobar

143 Insula_L (aal)

107 White Matter

98 Insula

60 Gray Matter

52 Extra-Nuclear

31 brodmann area 13

17 Claustrum

7 brodmann area 47

7 Inferior Frontal Gyrus

5 Frontal Lobe

4 Frontal_Inf_Tri_L (aal)

3 Putamen_L (aal)

2 brodmann area 45

----------------------

Cluster 6

Number of voxels: 98

Peak MNI coordinate: 38 34 24

Peak MNI coordinate region: // Right Cerebrum // Frontal Lobe // Middle Frontal Gyrus // White Matter // undefined // Frontal_Inf_Tri_R (aal)

Peak intensity: 1

# voxels structure

98 --TOTAL # VOXELS--

98 Frontal Lobe

98 Right Cerebrum

95 White Matter

86 Middle Frontal Gyrus

85 Frontal_Mid_R (aal)

13 Frontal_Inf_Tri_R (aal)

10 Sub-Gyral

3 brodmann area 9

3 Gray Matter

2 Superior Frontal Gyrus

----------------------

Cluster 7

Number of voxels: 50

Peak MNI coordinate: -44 -38 42

Peak MNI coordinate region: // Left Cerebrum // Parietal Lobe // Inferior Parietal Lobule // White Matter // undefined // Parietal_Inf_L (aal)

Peak intensity: 1

# voxels structure

50 --TOTAL # VOXELS--

50 Left Cerebrum

50 Parietal Lobe

38 Inferior Parietal Lobule

27 Postcentral_L (aal)

23 Gray Matter

23 White Matter

23 Parietal_Inf_L (aal)

22 brodmann area 40

8 Postcentral Gyrus

4 Sub-Gyral

1 brodmann area 2

----------------------

Cluster 8

Number of voxels: 163

Peak MNI coordinate: -4 8 46

Peak MNI coordinate region: // Left Cerebrum // Limbic Lobe // Cingulate Gyrus // Gray Matter // brodmann area 32 // Supp_Motor_Area_L (aal)

Peak intensity: 1

# voxels structure

163 --TOTAL # VOXELS--

148 Frontal Lobe

143 Supp_Motor_Area_L (aal)

134 Left Cerebrum

96 Medial Frontal Gyrus

82 Gray Matter

52 Superior Frontal Gyrus

49 White Matter

46 brodmann area 6

33 brodmann area 32

24 Right Cerebrum

18 Supp_Motor_Area_R (aal)

10 Limbic Lobe

10 Cingulate Gyrus

5 Inter-Hemispheric

2 brodmann area 24

1 brodmann area 8

----------------------

Cluster 9

Number of voxels: 65

Peak MNI coordinate: 28 -6 48

Peak MNI coordinate region: // Right Cerebrum // Frontal Lobe // Middle Frontal Gyrus // White Matter // undefined // Precentral_R (aal)

Peak intensity: 1

# voxels structure

65 --TOTAL # VOXELS--

65 Frontal Lobe

65 Right Cerebrum

58 Middle Frontal Gyrus

40 White Matter

25 brodmann area 6

25 Gray Matter

21 Frontal_Mid_R (aal)

20 Precentral_R (aal)

11 Frontal_Sup_R (aal)

7 Sub-Gyral

----------------------

Cluster 10

Number of voxels: 78

Peak MNI coordinate: -28 -6 52

Peak MNI coordinate region: // Left Cerebrum // Frontal Lobe // Middle Frontal Gyrus // White Matter // undefined // Frontal_Mid_L (aal)

Peak intensity: 1

# voxels structure

78 --TOTAL # VOXELS--

78 Frontal Lobe

78 Left Cerebrum

62 Middle Frontal Gyrus

55 Precentral_L (aal)

39 brodmann area 6

39 Gray Matter

34 White Matter

16 Frontal_Sup_L (aal)

12 Precentral Gyrus

7 Frontal_Mid_L (aal)

4 Sub-Gyral

>>

**NEEDING_ST_**

Threshold

-- p value = 1

-- intensity = -1.633123935319537e+16

-- cluster size = 5

Number of clusters found: 3

----------------------

Cluster 1

Number of voxels: 8

Peak MNI coordinate: 42 4 -12

Peak MNI coordinate region: // Right Cerebrum // Sub-lobar // Extra-Nuclear // Gray Matter // brodmann area 13 // Insula_R (aal)

Peak intensity: 1

# voxels structure

8 --TOTAL # VOXELS--

8 Insula_R (aal)

8 Right Cerebrum

5 Sub-lobar

5 Extra-Nuclear

4 brodmann area 13

4 Gray Matter

3 Sub-Gyral

3 Temporal Lobe

2 White Matter

----------------------

Cluster 2

Number of voxels: 92

Peak MNI coordinate: -36 -2 -10

Peak MNI coordinate region: // Left Cerebrum // Sub-lobar // Extra-Nuclear // White Matter // undefined // Insula_L (aal)

Peak intensity: 1

# voxels structure

92 --TOTAL # VOXELS--

92 Left Cerebrum

89 Sub-lobar

85 White Matter

77 Insula

71 Insula_L (aal)

9 Extra-Nuclear

7 Gray Matter

4 brodmann area 13

3 Sub-Gyral

3 Claustrum

3 Temporal Lobe

----------------------

Cluster 3

Number of voxels: 39

Peak MNI coordinate: 42 -6 -2

Peak MNI coordinate region: // Right Cerebrum // Sub-lobar // Insula // White Matter // undefined // Insula_R (aal)

Peak intensity: 1

# voxels structure

39 --TOTAL # VOXELS--

39 Sub-lobar

39 Right Cerebrum

22 White Matter

18 Extra-Nuclear

18 Insula_R (aal)

17 Gray Matter

14 Insula

12 Putamen_R (aal)

8 brodmann area 13

7 Claustrum

>>

**non-MID task meta-analysis**

A reviewer noted that “a very large majority of the task used to assess “wanting” consists in the MIDT…” and wondered if the same network would be observed even if the studies using the MIDT are removed? We ran a meta-analysis on the following ‘Wanting’_ST_ non-MID task articles (see the table below)

| Paper | Stimulus and Cue | Task description | Contrast | Healthy Participants |
| --- | --- | --- | --- | --- |
| Bradley, K. A., Case, J. A., Freed, R. D., Stern, E. R., & Gabbay, V. (2017). Neural correlates of RDoC reward constructs in adolescents with diverse psychiatric symptoms: A Reward Flanker Task pilot study. Journal of affective disorders, 216, 36-45. | Money | Reward Flanker Task | Reward Anticipation vs. Implicit Baseline | 22 |
| Richter, A., Petrovic, A., Diekhof, E. K., Trost, S., Wolter, S., & Gruber, O. (2015). Hyperresponsivity and impaired prefrontal control of the mesolimbic reward system in schizophrenia. Journal of psychiatric research, 71, 8-15. | Points, targets and CS | Desire-reason paradigm | desire context  reason context | 16 |
| Gluth, S., Rieskamp, J., & Büchel, C. (2013). Neural evidence for adaptive strategy selection in value-based decision-making. Cerebral Cortex, 24(8), 2009-2021. | Investment | dynamic learning task | Expected value | 24 |
| Trost, S., Diekhof, E. K., Mohr, H., Vieker, H., Krämer, B., Wolf, C., ... & Gruber, O. (2016). Investigating the impact of a genome-wide supported bipolar risk variant of MAD1L1 on the human reward system. Neuropsychopharmacology, 41(11), 2679. | Points, targets and CS | Desire-reason paradigm | desire context  reason context | 224 |
| Trost, S., Diekhof, E. K., Zvonik, K., Lewandowski, M., Usher, J., Keil, M., ... & Gruber, O. (2014). Disturbed anterior prefrontal control of the mesolimbic reward system and increased impulsivity in bipolar disorder. Neuropsychopharmacology, 39(8), 1914. | Points, targets and CS | Desire-reason paradigm | desire context | 16 |
| Yu, R., Mobbs, D., Seymour, B., Rowe, J. B., & Calder, A. J. (2014). The neural signature of escalating frustration in humans. Cortex, 54, 165-178. ISO 690 | cue, and coin | multi-trial reward schedule task | Cue – block  Cue (increased proximity)  Cue (increased expended effort) | 27 |
| Krebs, R. M., Schott, B. H., Schütze, H., & Düzel, E. (2009). The novelty exploration bonus and its attentional modulation. Neuropsychologia, 47(11), 2272-2281. | cue, reward | number comparison task (NCT) | reward-predicting cues in Exp 1: Contrast reward vs. neutral  familiar reward-predicting cues exp 1  reward-predicting cues in Exp 2: Contrast reward vs. neutral  novel reward-predicting cues exp 2 | 24 exp1  20 exp 2 |
| Articles from other sources |  |  |  |  |

We found peaks within the dopaminergic midbrain as well as the ventral and dorsal striatum. We also found a peak within the anterior insula.


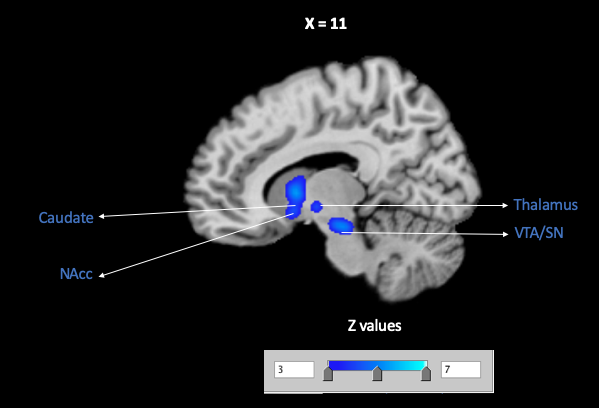


Thus, though experimental tasks for ‘Wanting’_ST_ included a lot of MID tasks, other included paradigms elicited (separately from the MID tasks) the same mesolimbic dopamine and ventral striatal network that has been related to incentive salience ‘Wanting’_ST_, although in terms of overall whole brain pattern they might have differed.
